# Identification of a novel CHN1 p.(Phe213Val) variant in a large Han Chinese family with congenital Duane retraction syndrome

**DOI:** 10.1038/s41598-020-73190-1

**Published:** 2020-10-01

**Authors:** Tai-Cheng Zhou, Wen-Hua Duan, Xiao-Lin Fu, Qin Zhu, Li-Yun Guo, Yuan Zhou, Zhi-Juan Hua, Xue-Jiao Li, Dong-Mei Yang, Jie-Ying Zhang, Jie Yin, Xiao-Fan Zhang, Guang-Long Zhou, Min Hu

**Affiliations:** 1 Department of Ophthalmology and Central Lab, The Second People’s Hospital of Yunnan Province, Kunming, Yunnan Province China; 2 Hainan Western Central Hospital, Danzhou, Hainan Province China

**Keywords:** Eye diseases, Disease genetics

## Abstract

Duane retraction syndrome (DRS) is a neuromuscular dysfunction of the eyes. Although many causative genes of DRS have been identified in Europe and the United States, few reports have been published in regard to Chinese DRS. The aim of the present study was to explore the genetic defect of DRS in a Chinese family. Exome sequencing was used to identify the disease-causing gene for the two affected family members. Ophthalmic and physical examinations, as well as genetic screenings for variants in chimerin 1 (*CHN1*), were performed for all family members. Functional analyses of a *CHN1* variant in 293T cells included a Rac-GTP activation assay, α2-chimaerin translocation assay, and co-immunoprecipitation assay. Genetic analysis revealed a NM_001822.7: c.637T > G variant in the *CHN1* gene, which resulted in the substitution of a highly conserved C1 domain with valine at codon 213 (NP_001813.1: p.(Phe213Val)) (ClinVar Accession Number: SCV001335305). *In-silico* analysis revealed that the p.(Phe213Val) substitution affected the protein stability and connections among the amino acids of CHN1 in terms of its tertiary protein structure. Functional studies indicated that the p.(Phe213Val) substitution reduced Rac-GTP activity and enhanced membrane translocation in response to phorbol-myristoyl acetate (PMA). Together with previous studies, our present findings demonstrate that *CHN1* may be an important causative gene for different ethnicities with DRS.

## Introduction

Duane retraction syndrome (DRS) is a congenital disorder that impairs eye movement^1^ and is caused by a failure of the sixth cranial nerve (i.e., the abducens nerve). DRS is characterized by a restriction or absence of abduction and/or adduction, partial closure of the eyelids, and globe retraction into the orbit upon adduction^1^. DRS is estimated to constitute 1–5% of all strabismus and to affect 0.1% of the general population^2^. Without treatment, DRS can result in amblyopia and blindness during childhood. Familial DRS is mainly inherited in an autosomal-dominant mode, which accounts for 10% of all DRS disorders^3^.

Based on the current understanding of the genetics of DRS, at least six genes—spalt like transcription factor 4 (*SALL4*), chimerin 1 (*CHN1*), homeobox A1 (*HOXA1*), MAF bZIP transcription factor B (*MAFB*), kinesin family member 21A (*KIF21A*), and tubulin beta 3 class III (*TUBB3*)—have been identified to be associated with DRS^4,5^. Duane retraction syndrome 2 (DURS2, OMIM 604356) and DURS3 (OMIM 617041) have been identified to be caused by variants in *CHN1* and *MAFB*, while DURS1 (OMIM 126800) maps to chromosome 8q13. Variants in *CHN1* hyper-activating α2-chimaerin are involved in familial non-syndromic DURS2 with incomplete penetrance^6^. *CHN1* can encode α2-chimaerin, which is a Rac guanosine triphosphatase (GTPase)-activating protein (RacGAP) that functions as a phorbol ester receptor and ras-related p21-rac that can be activated by upstream signaling in the plasma membrane^7–9^. Upon activation, Rac-GTP is hydrolyzed to inactive Rac-GDP via the α2-chimaerin GAP domain^10^, which alters oculomotor axons and extraocular muscle dynamics during the development of the chick embryo^6^.

Human populations with different ethnicities and from distinct geographical locations confer a diverse overall genetic background. More than 10 *CHN1* variants have been reported to be associated with DURS2 in Europe and the United States^6,15^. However, there are currently no reports regarding genetic abnormalities underlying DRS2 in a Chinese population. Hence, a further spectrum of variation for DRS is needed, particularly in Asian populations. The purpose of the present study was to screen for variants in DRS causal genes in a Han Chinese DRS family and to explore the functional mechanism of a novel *CHN1* substitution, NM_001822.7: c.637T > G, NP_001813.1: p.(Phe213Val) (ClinVar Accession Number: SCV001335305), underlying DRS2.

## Results

### Clinical phenotypes and laboratory analysis

**Figure 1:**
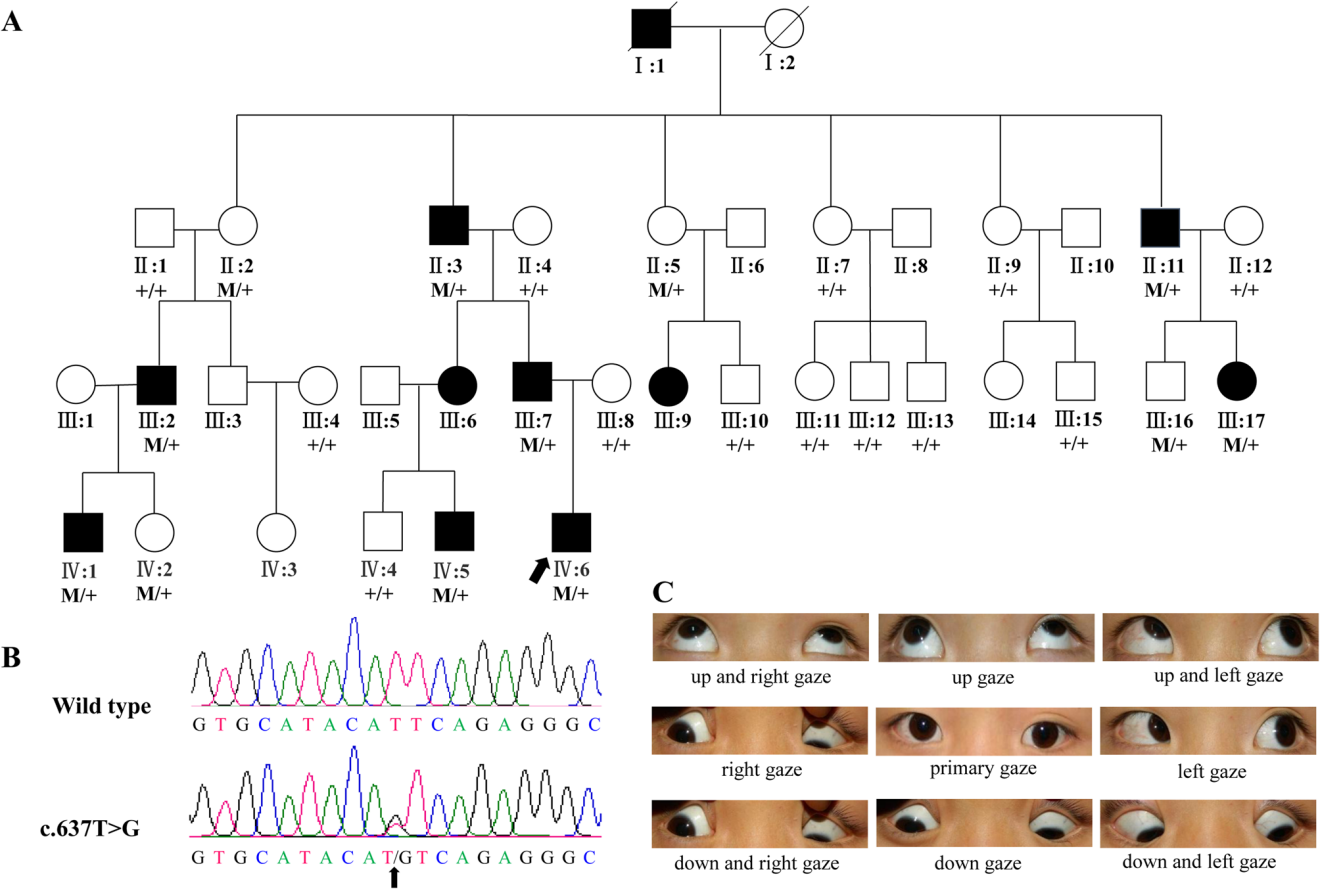
Pedigree of DRS family with a *CHN1* variant, sequencing chromatogram, and diagnostic positions of gaze analysis. (**A**) Pedigree of DRS family with a *CHN1* variant. The proband is marked by an arrow, black symbols denote affected members, white symbols denote unaffected members, squares denote males, and circles denote females. The presence or absence of the *CHN1* substitution (c.637T > G) is indicated by an ‘M’ or ‘+’, respectively. (**B**) Sequencing chromatogram. (**C**) Diagnostic positions of the gaze of subject IV:6. RefSeq of *CHN1*: NM_001822.7.

There were 37 members in the Han Chinese family who were considered for the present study, with 11 members being diagnosed with DRS during childhood. The DRS patients in this family were consistent with being representative of an incomplete dominant genetic model of DRS (Fig. 1A). We also evaluated the clinical phenotypes for 25 individuals in this family (Table 1). Among them, eight members were diagnosed with DRS and exhibited abnormal corneal optical reflections, squint angles, and globe retractions. Six family members (III:2, III:7, III:17, IV:1, IV:5, and IV:6) had bilateral DRS, whereas two individuals (II:3 and II:11) had left DRS. Interestingly, five of the DRS members were diagnosed with DRS type I, whereas one family member (III:2) was diagnosed with DRS type II. Additionally, two other family members (IV:1 and IV:5) had different DRS types in their left and right eyes.

**Table 1:** Features of DRS pedigree patients and unaffected relatives.

Subjects	Gender	Age	Substitution	UCVA		CVA		Corneal optical reflection	Squint angle (Δ)		Globe retraction	DRS type		
				OS	OD	OS	OD		33 cm	5 m		Left eye	Right eye	Affection
II: 1	M	54	+/+	0.8	0.5	1	1	Normal position			No			
II: 2	F	55	p.(Phe213Val)	0.8	0.6	1	1	Normal position			No			
II: 3	M	53	p.(Phe213Val)	0.8	0.8	1	1	R/L10Δ	R/L40Δ	R/L40Δ	Yes	Normal	DRS I	Right
II: 4	F	51	+/+	0.5	0.25	1	1	Normal position			No			
II: 5	F	48	p.(Phe213Val)	0.8	0.4	1	*	Normal position			No			
II: 7	F	46	+/+	0.8	1	1		Normal position			No			
II: 9	F	43	+/+	1	1			Normal position			No			
II: 11	M	40	p.(Phe213Val)	1	0.8	1	0.8	+ 5Δ(L)	+ 25Δ	+ 25Δ	Yes	DRS I	Normal	Left
II: 12	F	35	+/+	1	1			Normal position			No			
III: 2	M	33	p.(Phe213Val)	0.8	1	1		R/L	R/L5Δ	R/L5Δ	Yes	DRS I	DRS I	Bilateral
III: 4	F	24	+/+	1.2	1.2			Normal position			No			
III: 7	M	29	p.(Phe213Val)	0.02	1	*		+ 15Δ(L)	+ 50Δ	+ 50Δ	Yes	DRS I	DRS I	Bilateral
III: 8	F	26	+/+	1	0.8		1	Normal position			No			
III: 10	M	22	+/+	0.2	0.2	1	1	Normal position			No			
III: 11	F	26	+/+	0.6	0.5	1	1	Normal position			No			
III: 12	M	21	+/+	1	1			Normal position			No			
III: 13	M	16	+/+	0.5	0.3	1	1	Normal position			No			
III: 15	M	21	+/+	1	1			Normal position			No			
III: 16	M	16	p.(Phe213Val)	1	1			Normal position			No			
III: 17	F	11	p.(Phe213Val)	1	0.4		0.6	+ 10Δ(R)	+ 60ΔR/L10Δ	+ 60ΔR/L10Δ	Yes	DRS I	DRS I	Bilateral
IV: 1	M	13	p.(Phe213Val)	0.8	0.4	1	0.5	− 10Δ(R)	− 40ΔL/R	− 40Δ	Yes	DRS I	DRS III	Bilateral
IV: 2	M	12	p.(Phe213Val)	0.5	0.8	0.8	1	Normal position			No			
IV: 4	M	10	+/+	1	1			Normal position			No			
IV: 5	M	8	p.(Phe213Val)	0.5	0.8	0.6	0.8	Normal position			Yes	DRS II	DRS I	Bilateral
IV: 6	M	8	p.(Phe213Val)	0.6	1	0.8		+ 10Δ(R)	+ 30Δ	+ 30Δ	Yes	DRS I	DRS I	Bilateral

*UCVA* uncorrected visual acuity, *CVA* corrected visual acuity, *DRS* Duane retraction syndrome.

*Correction without improvement, OS: left eye, OD: right eye, +/+: wild type, RefSeq of CHN1: NP_001813.1.

One family member (IV:6) was diagnosed with bilateral DRS as a proband (Fig. 1C). He exhibited bilateral horizontal-rectus muscle co-contraction and a bilateral-exodeviation limitation. The abduction limitation was mild in the right eye and moderate in the left eye. His corrected visual acuities of the right eye and left eye were each 1.0 logMAR. The results of the cornea/lens examination and fundus examination were each normal. Magnetic resonance imaging was not obtained. The degree of strabismus was + 75Δ, and stereopsis was deficient. The patient then underwent strabismus surgery. After surgery, his eye position was notably improved.

Another family member (IV:5) was diagnosed as having bilateral DRS (Supporting Information Figure S1). His bilateral eyes were mildly exodeviated. He had a history of a left head tilt since early childhood. His corrected visual acuities of the right eye and left eye were each 0.9 logMAR. The results of the cornea/lens examination and fundus examination were each normal. Magnetic resonance imaging was not obtained. Further examination revealed right hypertropia, as well as a large deorsumversion deficit. The degree of strabismus was R\L30Δ, and stereopsis was deficient. After surgery, both his left head tilt and eye position were notably improved.

### Genetic analysis

DNA samples from 25 living family members from the included Han Chinese family were used for genetic studies and were comprised of samples from nine DRS patients and 16 unaffected family members. In addition, we collected 200 DNA samples from ethnically matched controls to verify *CHN1* variants. Samples from two family members (III:11 and III:17) were used for exome sequencing to screen for variants. After filtering procedures (Tables S1 and S2), we identified a novel non-synonymous potential disease-associated variant (NM_001822.7: c.637T > G, NP_001813.1: p.(Phe213Val)) in *CHN1* (i.e., the gene encoding α2-chimaerin). Based on a dominant model, we selected rare variants to perform co-segregation analysis for this pedigree. This variant co-segregated with all DRS family members and four individuals whose eyes were clinically normal (Fig. 1A and Table 1).

In this variant, valine was substituted for phenylalanine at position 213 in CHN1 (p.(Phe213Val)), which was not yet present in the 1000 Genomes Project dataset (https://browser.1000genomes.org/index.html). This variant was also not detected in the 200 ethnically matched controls from the same Southwestern Chinese population (data not shown). We also sequenced all coding exons and splice sites of *CHN1*; however, we did not detect any suspicious variations other than the c.637T > G substitution.

**Figure 2:**
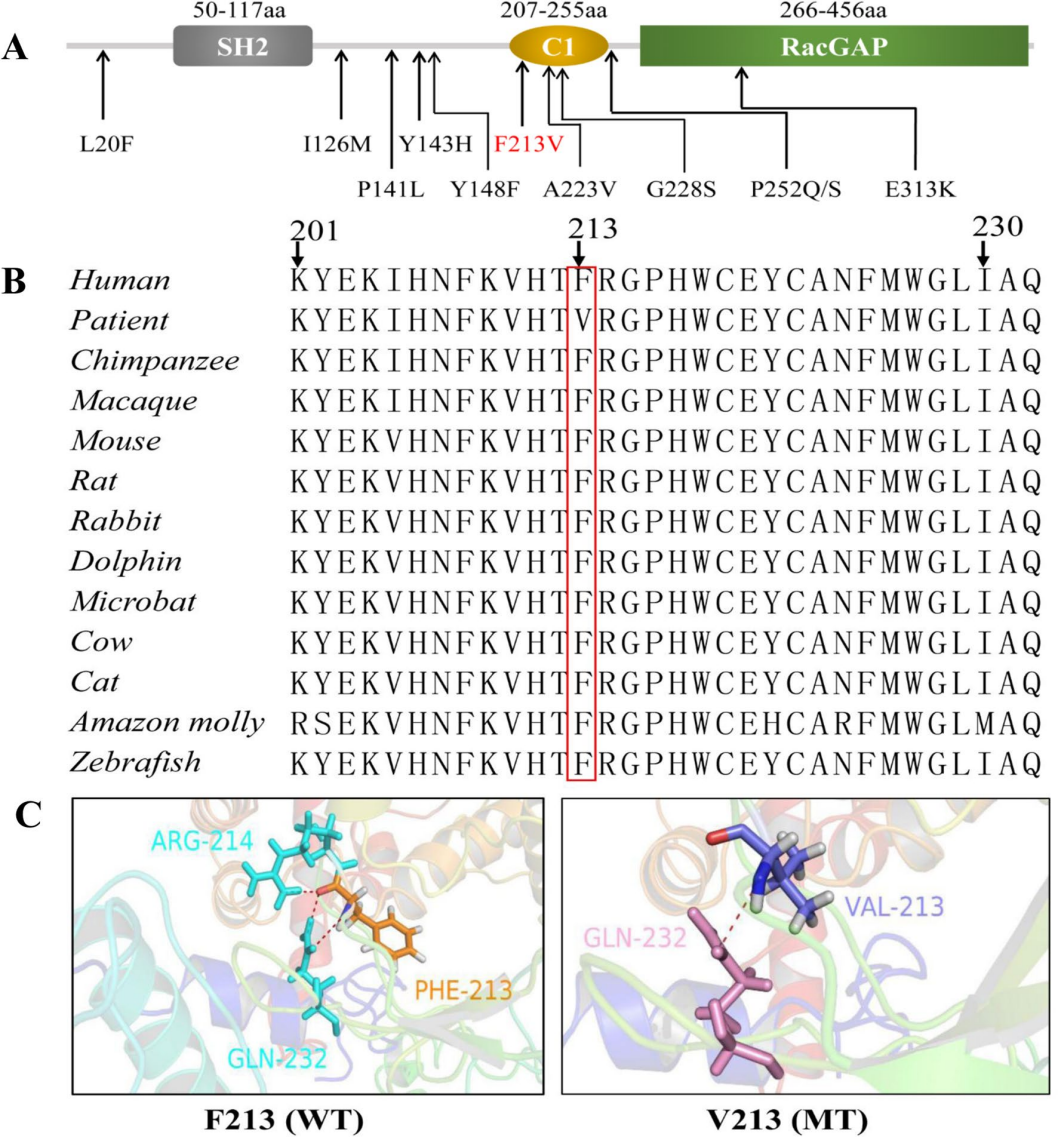
*CHN1* variant analysis. (**A**) Two-dimensional representations of the α2-chimaerin protein. The protein had three domains: C1, Rac-GAP, and Src homology 2 (SH2). The novel c.637T > G substitution altered residue F213 (p.(Phe213Val)), which was present in the C1 domain of α2-chimaerin (red) and was within a coil structure and a PKC phosphorylation site. Previously reported variants are also indicated. (**B**) The α2-chimaerin protein alignment among 12 different species demonstrates the evolutionary conservation of residue F213 (boxed in red). (**C**) Homology modeling of the CHN1 protein shows the conformational changes induced with and without the p.(Phe213Val) substitution. RefSeq of *CHN1*: NM_001822.7.

The p.(Phe213Val) substitution mapped to the C1 domain of CHN1, which was within its coil structure (Supporting Information Figure S2) and was located in a PKC phosphorylation site (PS00005). The p.(Phe213Val) substitution led to a highly conserved amino-acid change, as indicated by evolutionary-conservation analysis (Fig. 2). The results of MutationTaster, Sorting Tolerant From Intolerant (SIFT), and Polymorphism Phenotyping v2 (PolyPhen-2) predicted that the p.(Phe213Val) substitution had a disease-causing effect. Protein-function prediction suggested that the F213 in the wild-type CHN1 protein was associated with two other residues (R214 and Q232) via three connecting hydrogen bonds, while the substitution that we identified for CHN1 (V213) led to one connecting hydrogen bond with residue Q232. This replacement was predicted to result in the loss of two structuring H-bonds, introducing conformational changes in the affected region. For computational-stability analysis, we used the three-dimensional protein structure of human CHN1 (PBD ID: 3CXL) to assess the stability of the p.(Phe213Val) variant. The results were consistent with those of nine other variants that have been reported previously (Table 2); specifically, p.(Phe213Val) induced notable destabilization with a large decline in Gibbs free energy, as indicated by negative ΔΔG values determined by four different types of software.

**Table 2:** Prediction of protein-stability changes due to single amino-acid substitutions in CHN1.

AA change	Inheritance	I-Mutant 2.0	mCSM	SDM	DUET	Prediction
L20F	AD	0.26	− 1.695	− 1.39	− 1.95	Destabilizing
I126M	AD	− 0.35	− 1.066	− 0.52	− 1.01	Destabilizing
P141L	AD	− 0.61	− 0.807	2.74	0.067	–
Y143H	AD	− 0.03	− 1.526	− 0.97	− 1.421	Destabilizing
Y148F	AD	− 0.33	− 1.118	0.45	− 0.707	Destabilizing
F213V	AD	− 3.09	− 1.89	− 2.33	− 2.13	Destabilizing
A223V	AD	− 0.28	− 0.383	0.1	− 0.166	Destabilizing
G228S	AD	− 0.87	− 1.464	− 3.47	− 1.789	Destabilizing
P252Q	AD	− 1.13	− 1.123	0.13	− 0.763	Destabilizing
P252S	AD	− 0.61	− 1.456	0.45	− 1.041	Destabilizing
E313K	AD	− 2.23	− 0.893	− 0.77	− 0.703	Destabilizing

Two-stage prediction classification: destabilizing (< 0 kcal/mol), stabilizing (> 0 kcal/mol); *AD* autosomal dominant; *AA* amino acid; predicted results were decided by more than two software analyses; RefSeq of CHN1: NP_001813.1.

### Functional analysis of the p.(Phe213Val) amino-acid substitution

To verify potential effects of the p.(Phe213Val) substitution on CHN1 function, we performed overexpression of variant or wild-type *CHN1* to induce differential Rac-GTP activation, α2-chimaerin translocation, and self-assembly.

**Figure 3:**
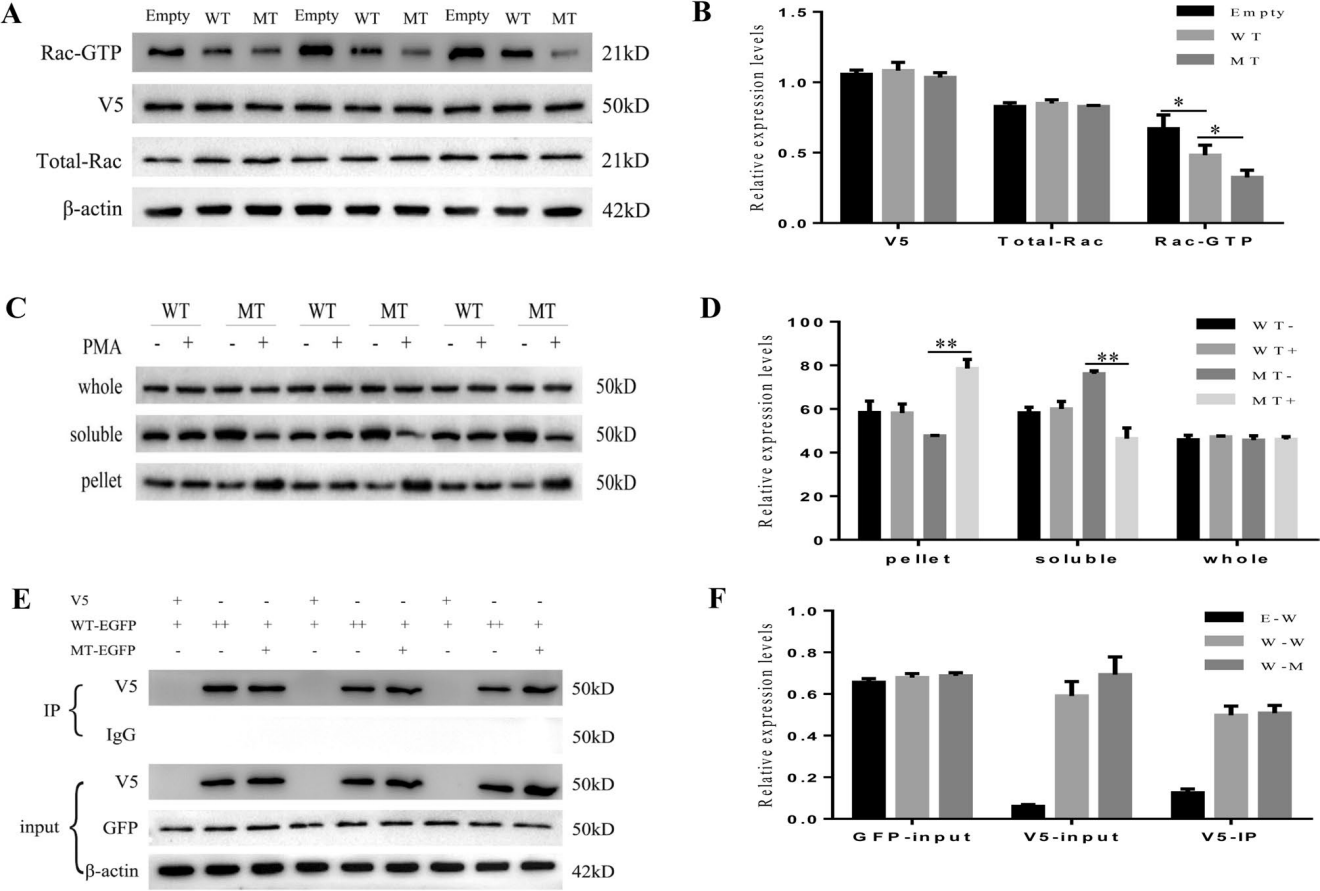
Functional studies of the novel c.637T > G *CHN1* substitution (MT) compared with wild-type (WT) *CHN1*. (**A**) Western blotting analysis of Rac-GTP, V5, total Rac, and β-actin. (**B**) Grayscale results of Western blotting. Top blot: WT α2-chimaerin reduced Rac-GTP levels compared to those with the empty vector, and MT α2-chimaerin lowered these levels below those of WT. The levels of total Rac (second blot) and V5-α2-chimaerin (third blot) in all groups were equal, as were the levels of β-actin (bottom blot) (n = 3). (**C**) Western-blotting analysis of α2-chimaerin from the PMA-stimulated α2-chimaerin membrane-translocation experiment and (**D**) grayscale results of Western blotting. Although total α2-chimaerin was comparable in all conditions, PMA stimulated translocation of α2-chimaerin into the pelleted (membranous) fraction. MT α2-chimaerin translocation was enhanced compared to that of WT (n = 3). (**E**) Western blotting showing a slight increase in the self-assembly of WT α2-chimaerin with MT α2-chimaerin compared to self-assembly of WT with WT α2-chimaerin (n = 3) and (**F**) Grayscale results of Western blotting. Abbreviations are as follows: −: non-transfected vector, +: transfected vector WT: wild type, MT: variant type, E-W: empty vector and wild type vector, W-W: wild type vector and wild type vector, M-W: variant type vector and wild type vector. RefSeq of CHN1: NP_001813.1.

We used a Rac-GTP activation assay to analyze Rac-GTP levels. Compared with that of an empty vector, overexpression of wild-type *CHN1* lowered Rac-GTP levels, which is consistent with the known function of α2-chimaerin. Moreover, overexpression of variant *CHN1* further reduced Rac-GTP levels compared to that of wild-type *CHN1* (Fig. 3A,B, and Supporting Information).

To assess the influence of the p.(Phe213Val) substitution on protein translocation from the cytoplasm to the plasma membrane, we quantified wild-type and variant α2-chimaerin in pelleted and soluble fractions prior to or after stimulation with PMA. As predicted, after PMA stimulation, variant α2-chimaerin was significantly increased in the soluble fraction, whereas it was concomitantly decreased in the pelleted fraction. For wild-type α2-chimaerin, there was no difference in the proportion of α2-chimaerin in soluble/pelleted fractions prior to and after stimulation with PMA (Fig. 3C,D, and Supporting Information). These results suggest that this α2-chimaerin variant induced aberrant translocation to the plasma membrane after PMA stimulation.

We also tested if variant hyper-activated α2-chimaerin could interact with the wild-type protein to recruit wild-type α2-chimaerin to the plasma membrane, which may further reduce Rac activity. In the presence of PMA, the immunoprecipitation of wild-type α2-chimaerin with variant α2-chimaerin was slightly enhanced compared to its interaction with itself, but this difference was not statistically significant (Fig. 3E,F, and Supporting Information).

Taken together, the p.(Phe213Val) substitution reduced Rac-GTP activity and enhanced translocation to the plasma membrane by putatively changing the conformation of α2-chimaerin in response to PMA, without affecting protein self-assembly.

### American College of Medical Genetics (ACMG) evaluation of the *CHN1* c.637T > G (p.(Phe213Val)) substitution

Conforming to the classification of the American College of Medical Genetics (ACMG) for evaluating the pathogenicity of different variants, the p.(Phe213Val) substitution of CHN1 was “pathogenic” as it was well-established via in-vitro functional studies supportive of a deleterious effect on the CHN1 protein (PS3) in a PKC phosphorylation site (PM1) that was absent in population databases (PM2), and there was cosegregation with affected family members (PP1), computational evidence showed a damaging effect (PP3), and a highly specific disease phenotype (DRS) with via a single gene (*CHN1*) (PP4). Thus, according to the ACMG evaluation, the p.(Phe213Val) substitution of CHN1 had one strong (PS3), two moderate (PM1, PM2), and more than two supportive (PP1, PP3, PP4) findings of pathogenicity, meeting the criteria of the ACMG for a substitution to be considered a pathogenic variant.

## Discussion

Patients with DRS have globe retraction upon adduction, which is due to the absence of normal functioning of abducens motor neurons and the abducens nerve to regulate the lateral rectus muscle^11^; hence, DRS involves abnormal neuromuscular regulation of oculomotor function. DRS is divided into three types (DRS type I, DRS type II, and DRS type III) based on different muscular limits (abduction, adduction, or both, respectively) according to the Huber’s classification system^12^. All three types of DRS were detected in our pedigree, which is consistent with previous observations that have suggested phenotypic diversity of DRS^12,13^. According to genetic heterogeneity, DURS1 maps to chromosome 8q13, whereas DURS2 and DRS3 are caused by variants in the *CHN1* gene and *MAFB* gene, respectively^5,11,14^.

*CHN1* is one of the most important and common pathogenic genes for DRS, and nearly 10 variants (L20F, I126M, P141L, Y143H, Y148F, A223V, G228S, P252Q/S, and E313K) have already been reported (Fig. 2A)^6,15,16^. Previous studies on the functional analyses of these *CHN1* variants have revealed that p.F213V-, L20F-, Y143H-, Y148F-, A223V-, and P252Q-α2-chimaerin mutant proteins can enhance Rac-GAP activity and membrane translocation by destabilizing the closed conformation of α2-chimaerin in response to PMA^6,15,16^. Additionally, in the presence of PMA, previous studies have demonstrated that the interaction of wild-type-α2-chimaerin with mutants (L20F, I126M, Y143H, Y148F, A223V, and P252Q) is significantly enhanced compared to its interaction with itself, but that there is no effect regarding the interactions of variants of α2-chimaerin (G228S and E313K)^6,15,16^.

These variants have primarily been detected in non-Chinese individuals^6,15,16^. In the present study, we reported a novel p.(Phe213Val) substitution in a large Han Chinese family with reduced penetrance to congenital DRS. We used exome sequencing and bioinformatic analysis to identify a specific p.(Phe213Val) substitution in CHN1, which was carried by all affected members in our pedigree. The p.(Phe213Val) substitution is a rare CHN1 variant that was not found in either existing databases or in the 200 ethnically matched controls analyzed in the present study. The p.(Phe213Val) substitution is a conserved amino-acid substitution (Fig. 2) and is located in a PKC phosphorylation site (PS00005), which may change its phosphorylated state. The p.(Phe213Val) substitution reduced connections between amino acids of CHN1 in terms of its tertiary protein structure (Fig. 2B). These changes were also confirmed via mutation-stability analysis.

Mutation-stability analysis was executed for the p.(Phe213Val) substitution and for the 10 other related DRS variants in CHN1 that have been reported in previous studies^6,15,16^. Aside from the p.P141L substitution, the p.(Phe213Val) substitution and the other nine variants were all classified as destabilizing in terms of exhibiting declines in Gibbs free energy (Table 2). Interestingly, among these variants, the p.(Phe213Val) substitution was the most unstable, as indicated by its ΔΔG value =  − 2.13 via DUET software analysis. This result was consistent with the high conservation values of aa.F213, which suggests an essential contribution of aa.F213 to functional and/or structural CHN1 stability.

Based on the above analysis, we speculated that the p.(Phe213Val) substitution would induce hyper-activated α2-chimaerin RacGAP activity by destabilizing its closed conformation. In our present study, in response to PMA, α2-chimaerin with the p.(Phe213Val) substitution enhanced RacGAP activity and membrane translocation, which may have been induced by substitution-mediated opening of the closed conformation of CHN1. Interestingly, p.L20F-, Y143H-, Y148F-, A223V-, and P252Q-α2-chimaerin mutant proteins appear to enhance membrane translocation and RacGAP activity by destabilizing the closed conformation of α2-chimaerin in response to PMA, as indicated by results from previous studies^6,15^.

However, in our present study, in the presence of PMA, the interaction of wild-type-α2-chimaerin with mutants (L20F, I126M, Y143H, Y148F, A223V, and P252Q) was significantly enhanced compared to its interaction with itself; however, variants (G228S and E313K) had no such changes in their interactions with each other^6,15^. Consistent with the results of the interactions between mutants (G228S and E313K), we detected that the interaction between the p.(Phe213Val) substitution and wild-type-α2-chimaerin was not significantly enhanced. This may account for the reduced penetrance found in this pedigree, which would be consistent with the known incomplete penetrance in G228S and E313K variant pedigrees. Taken together, our findings suggest that α2-chimaerin with p.F231V can enhance membrane translocation and RacGAP activity.

In the family analyzed in the present study, the p.F231V in CHN1 excessively activated α2-chimaerin via enhancing α2-chimaerin’s membrane translocation and activity, hydrolyzing Rac-GTP, and altering oculomotor axons and extraocular muscle dynamics, all of which led to the DURS2 phenotype. To the best of our knowledge, the present study is the first to report that a substitution in the *CHN1* gene is involved in the pathogenesis of DRS in a Chinese family. Combined with the results of previous studies, our present study corroborates that *CHN1* plays an important role in the pathogenesis of DRS in humans.

## Materials and methods

### Ethical considerations

This study was approved by the Clinical Research Ethics Committee of the Second Hospital of Yunnan Province (2019098) and adhered to the Declaration of Helsinki Principles. Written informed consent for both study participation and publication of identifying information/images in an online open-access publication was obtained from all participants or their parents (for participants under the age of 18 years).

### Human subjects

A four-generation Han Chinese family diagnosed with autosomal incompletely dominant congenital DRS was enrolled in the present study at the Second People’s Hospital of Yunnan Province (Kunming, China). This family included 34 subjects comprised of 10 affected members and 24 unaffected members. In the present study, we recruited 25 of these family members, which included eight affected members (Fig. 1). The 200 ethnically matched controls had normal eyes and were also enrolled at the Second People’s Hospital of Yunnan Province (Kunming, China).

All 25 family members underwent physical and ophthalmic examinations, which included tests of vision, slit-lamp biomicroscopy, and fundus examination during pupil dilation, as well as measurements of diopter, degree of strabismus, eye movement, and corneal curvature. These tests and measurements were used to identify whether there were any other ocular or systemic abnormalities present in the included family members (Table 1).

### Exome sequencing and data analysis

Genomic DNA samples were extracted from peripheral blood of DRS patients and unaffected family members. We performed exome sequencing on family members (III:11 and III:17; Fig. 1A). Exome sequencing and data analysis were conducted according to a previous study^17^ (Tables S1 and S2). Exonic variants—including missense variants, stop-codon variants, splice-site variants, and small frameshift and non-frameshift insertions and deletions (INDELs)—were filtered by ExAC, dbSNP, 1000 Genomes, and the ClinSeq database, based on a minor allele frequency below 1%. We also excluded synonymous variants within intergenic, intronic, and untranslated regions (UTRs). These variants were further filtered based on a heterozygous inheritance model. Moreover, the software packages, SIFT (https://sift.bii.astar.edu.sg/) and PolyPhen-2 (https://genetics.bwh.havard.edu/pph2), were used to evaluate potentially damaging effects of the variants on protein structure/function. Four online servers (I-Mutant 2.0, Site-Directed Mutator (SDM), mCSM and DUET)^18–21^, based on Gibbs free energy (indicated by a ΔΔG value), were used to assess the stability of each variant. The variations in ΔΔG were as follows: destabilizing (< 0 kcal/mol) and stabilizing (> 0 kcal/mol). The protein structure was predicted using I-TASSER. The PyMOL Molecular Graphics System (version 1.8; Schrödinger, LLC, New York City, NY, USA) was employed to determine functional changes in proteins in response to variants.

### Sanger sequencing

*CHN1* (RefSeq NM_001822.7) gene-variant analysis was performed by sequencing of the coding exons and the exon–intron boundaries of the *CHN1* gene. Primers were designed according to the positions of coding exons, following the protocols of a previous study^22^. PCR amplification was performed in a 40-μL reaction mixture containing 50–100 ng of DNA, 2 mM of dNTP, 2 μM of each forward and reverse primer, 1 × LA Taq PCR buffer, and two units of LA Taq polymerase (TaKaRa, Japan). The PCR conditions were as follows: 95 °C for 5 min; 40 cycles of 95 °C for 30 s, 60 °C for 30 s, and 72 °C for 30 s; and a final extension of 72 °C for 5 min. Purified PCR products were directly sequenced using PCR primers and the BigDye Terminator v3.1 Cycle Sequencing Kit (Applied Biosystems, CA, USA) on a 3730XL DNA sequencer (Applied Biosystems). DNASTAR SeqMan software (DNASTAR Inc, Madison, WI, USA) was used to analyze DNA sequences. Most of the family members and 200 unrelated control subjects from the same Southwestern Chinese population were sequenced to confirm gene variants. Newly determined sequences from the family members were deposited into GenBank (Accession numbers: MT919950-MT919974).

### Functional analysis

#### Rac-GTP activation assay

Three constructs (V5 empty, V5 wild-type CHN1, and V5 variant p.(Phe213Val) CHN1) were constructed based on the pCDNA3.1 (+) vector and were transiently transfected into the human embryonic kidney (HEK) 293T cells. HEK293T cells were cultured at 37 °C in an incubator with 10% CO_2_. Cells were cultured in Dulbecco’s modified Eagle’s medium (DMEM) (Invitrogen, USA) supplemented with 10% fetal bovine serum (Gibco, USA), 1% glutamine, and 1% of a double-antibody. After 48 h in culture, a Rac-GTP activation assay was conducted using a Rac1-activation assay kit (Abcam, Cambridge, MA, USA), which was performed according to the manufacturer’s instructions. Briefly, cells were solubilized in lysis buffer. PAK1 PBD agarose beads were used to selectively pull-down active Rac from protein extracts. Subsequently, the precipitated GTP-Rac was detected via Western blotting using a mouse anti-Rac1 specific monoclonal antibody (BD Transduction Laboratories, San Jose, CA). β-actin was also detected—which was used as an internal reference—using β-actin mAB (Abmart, P3002, Arlington, MA, USA) and HRP-conjugated goat Anti-rabbit IgG (CST, 7074, USA). Secondary antibodies and signals were detected with an ECL detection system.

#### Quantification of α2-chimaerin translocation

After 48 h of transfection with wild-type and variant V5 constructs, HEK293T cells were pretreated with 5 μM of bisindolylmaleimide I (LC Laboratories, Woburn, MA) for 30 min to inhibit protein kinase C (PKC). Then, cells were divided into the following two groups (each group divided into six dishes): the first group was subjected to 10 μM of PMA (Sigma, Cambridge, MA, USA) stimulation for 20 min; and the other group was used as a control without stimulation. Next, three dishes of cells for each group were used for Western blotting. Other cells from each group were sonicated in CSK buffer^23^ and centrifuged at 100,000*g* at 4 °C for 1 h to separate the soluble and pelleted fractions (i.e., the membranous and cytoskeletal components). Each pellet was then resuspended in an equal volume of CSK buffer. Western blotting was performed with a goat polyclonal anti-V5 antibody (Abcam, ab9137, Cambridge, UK) and an HRP-conjugated rabbit Anti-goat IgG (Abcam, ab6885, USA).

### Co-immunoprecipitation experiments

The GFP-fused wild-type_CHN1 vector was constructed base on the GV230 plasmid (GeneChem Co., Ltd., Shanghai, China). Specifically, 3 μg of V5 empty vector, V5 wild-type_CHN1 vector, or V5 variant_CHN1 vector was transiently co-transfected with 3 μg of GFP-fused wild-type _CHN1 vector into HEK293T cells. After 48 h of incubation, HEK293T cells were pretreated with 5 μM of a PKC inhibitor for 30 min and were then treated with 10 μM of PMA (Sigma, Cambridge, MA, USA) for 20 min. After 30 min of treatment on ice with lysis buffer, the cells were centrifuged at 18,000*g* at 4 °C for 20 min. The supernatant was precipitated using a rabbit polyclonal anti-GFP antibody (ab6556; Abcam, Cambridge, UK), and the pellet was then transferred to a nitrocellulose membrane. The signal was detected by a goat polyclonal anti-V5 antibody and an HRP-conjugated rabbit anti-goat IgG (Abcam, ab6885, USA). β-actin was also detected, as described in an earlier methods subsection, and was used as an internal control.

### Statistical analysis

The grayscale results of Western blotting were used for semiquantitative analysis. Statistical differences were assessed with unpaired Student’s *t*-tests using GraphPad Prism 6 software. Differences were considered statistically significant at *P* values less than 0.05.

## Supplementary information

Supplementary Information.
