# Supplementary material for: Identification of a novel CHN1 p.(Phe213Val) variant in a large Han Chinese family with congenital Duane retraction syndrome
Source: Sci Rep. 2020 Oct 1;10:16225. doi: 10.1038/s41598-020-73190-1 (PMC7531002; doi:10.1038/s41598-020-73190-1)
Supplement: Supplementary file 1 — Supplementary Information. [file 41598_2020_73190_MOESM1_ESM.pdf]

Supplementary Information

Identification of a novel CHN1 p.(Phe213Val) variant in a large Han Chinese family with congenital Duane retraction syndrome

Tai-Cheng Zhou[a,#], Wen-Hua Duan[a,#], Xiao-Lin Fu[b,#], Qin Zhu[a], Li-Yun Guo[a], Yuan Zhou[a], Zhi-Juan Hua[a], Xue-Jiao Li[a], Dong-Mei Yang[a], Jie-Ying Zhang[a], Jie Yin[a], Xiao-Fan Zhang[a], Guang-Long Zhou[a], Min Hu[a,*]

[a]Department of Ophthalmology and Central Lab, the Second People's Hospital of Yunnan Province, Kunming, Yunnan Province, China.

[b]Hainan Western Central Hospital, Danzhou, Hainan Province, China.

[#]These authors contributed equally to this work.

*Address for correspondence and reprints:

Dr.Min Hu, The Ophthalmology Department of the Second People’s Hospital of Yunnan Province, Kunming 650021, Yunnan, China.E-mail: fudanhumin123@sina.com.

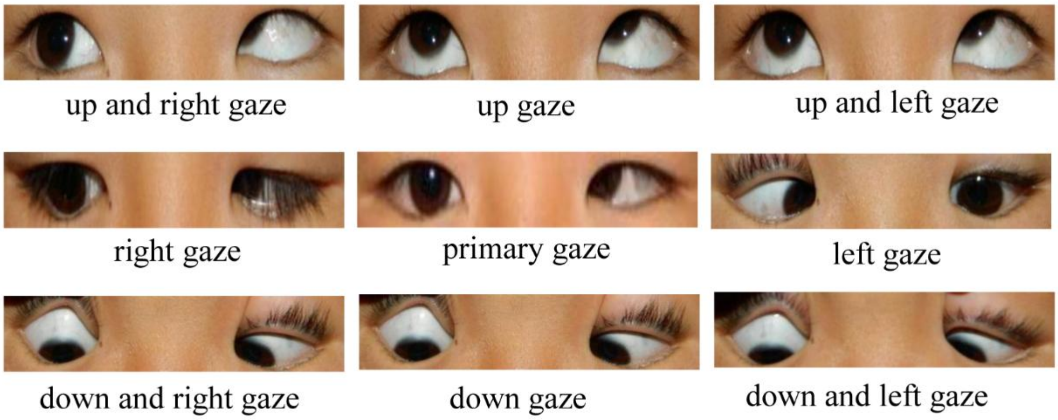

Supporting Information Figure S1 Diagnostic positions of the gaze of subject IV:5.

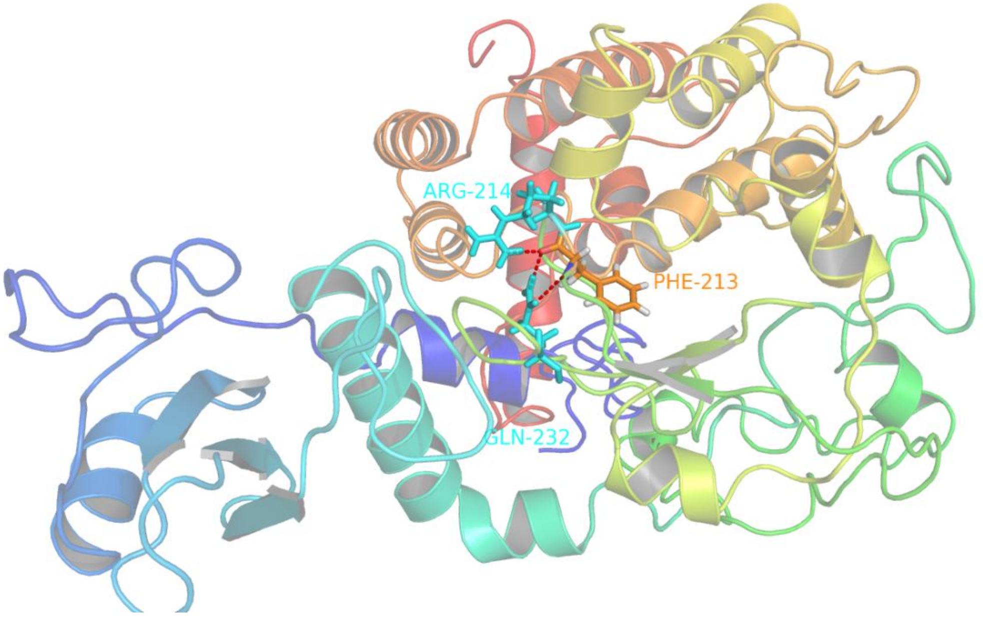

Supporting Information Figure S2 Homology modeling of the CHN1 protein with wild-type F213 and its connected amino acids (R214 and Q232).

Table S1 Filtering procedure of variants obtained by exome sequencing data analysis.

	III :11	III :17
Total variants	79684	79586
QC and classification filter	19187	18970
Genotype filter	4824	
Allele-frequency filter	508	
Variant-types filter	269	
Functional prediction	4	
Validation (segregation)	1	

Note: The number indicates an amount of variants passed for each step

Table S2 Candidate variants from exome sequencing that passed the validation process.

Position	Gene name	Variant type	Amino acid changes	rsID 142	SIFT	PolyP-hen2	OMIM Number	OMIM Disorder
chr11:17132074	*PIK3C2A*	nonsynonymous SNV	PIK3C2A:NM_002645:exon21:c.T3449C:p.L1150S	rs144811457	D	D	603601	-
chr2:105984191	*FHL2*	nonsynonymous SNV	FHL2:NM_001450:exon4:c.C337T:p.R113C	rs140148322	D	D	602633	-
**chr2:175689237**	***CHN1***	**nonsynonymous SNV**	**CHN1:NM_001822:exon8:c.T637G:p.F213V**	**-**	**D**	**D**	**118423**	**Duane retraction syndrome 2**
chr19:49100158	*SULT2B1*	nonsynonymous SNV	SULT2B1:NM_004605:exon5:c.G763A:p.G255R	-	D	D	604125	-

Note: The variants that passed the segregation process are presented with bold letters.

Supporting Information data The original images of gels and blots for Western blotting analysis are shown.

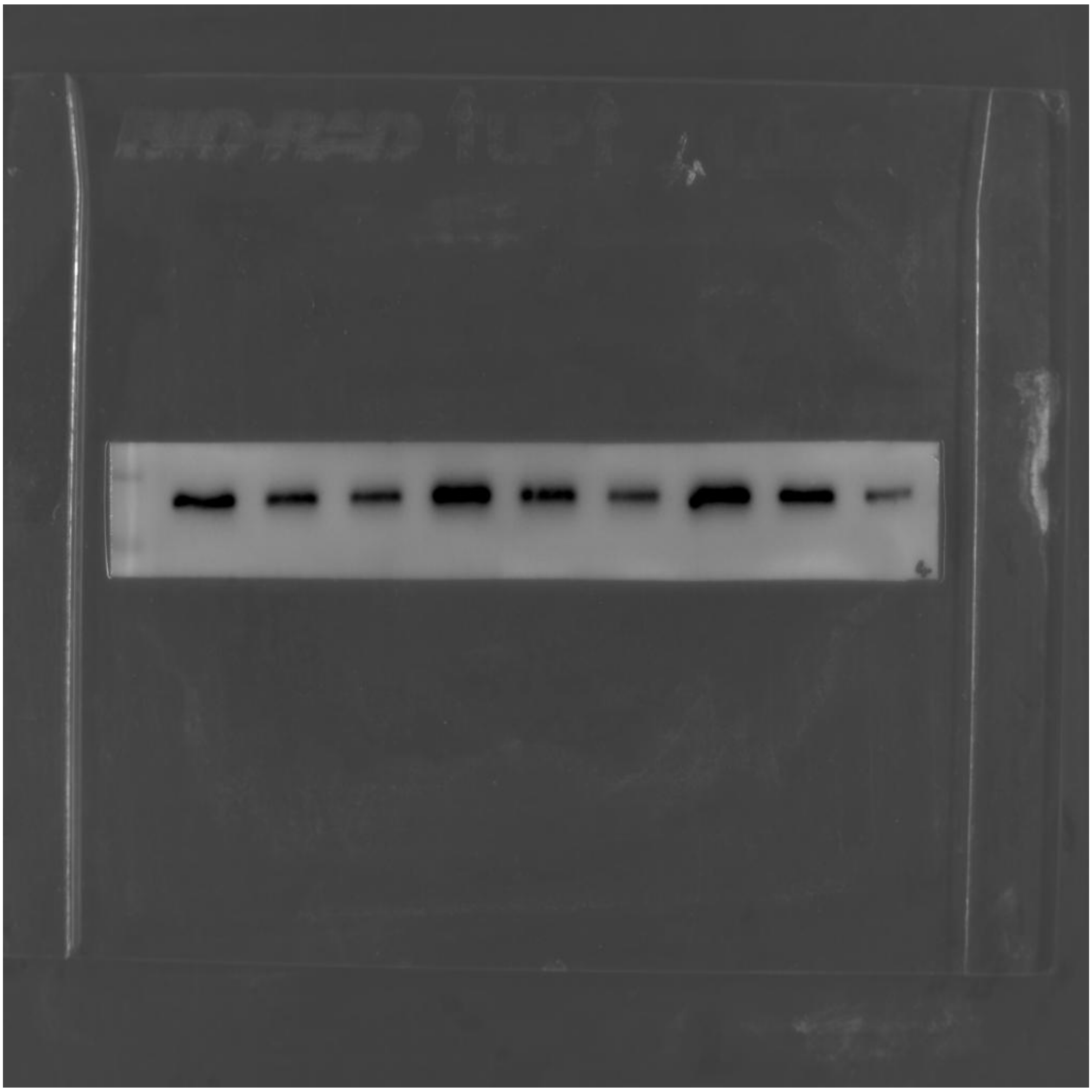

Figure 3A- Rac-GTP

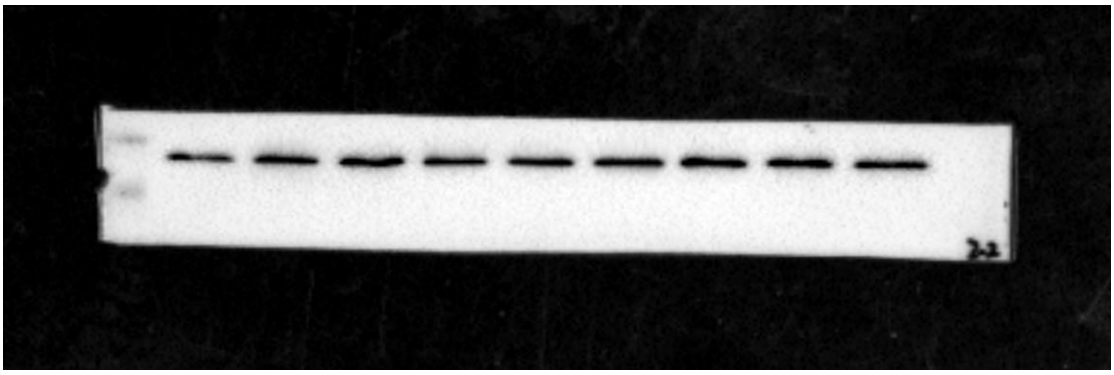

Figure 3A-Total-Rac

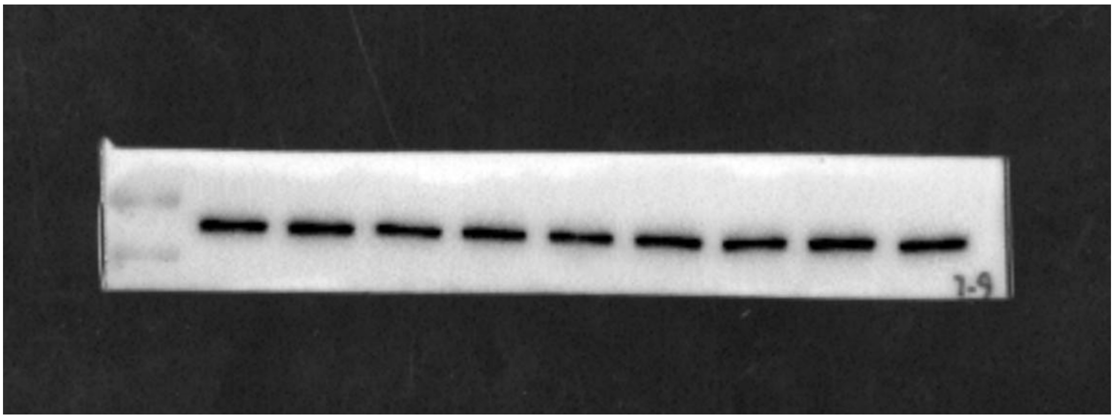

Figure 3A-V5

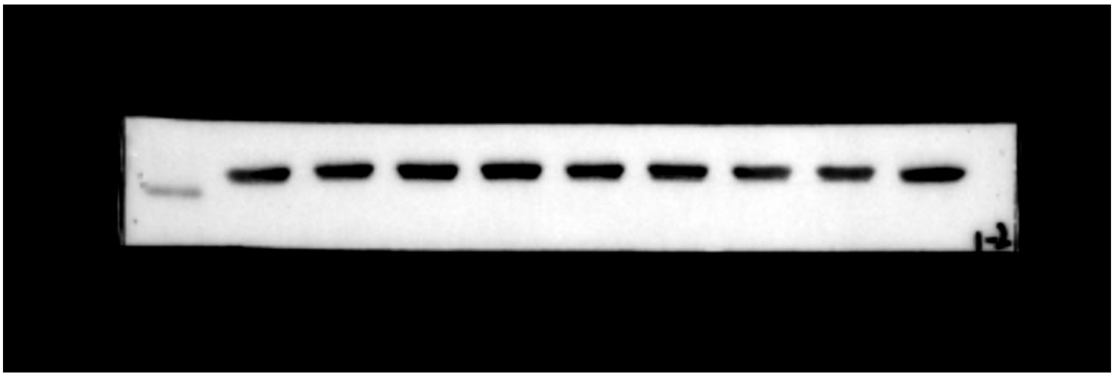

Figure 3A-β-actin

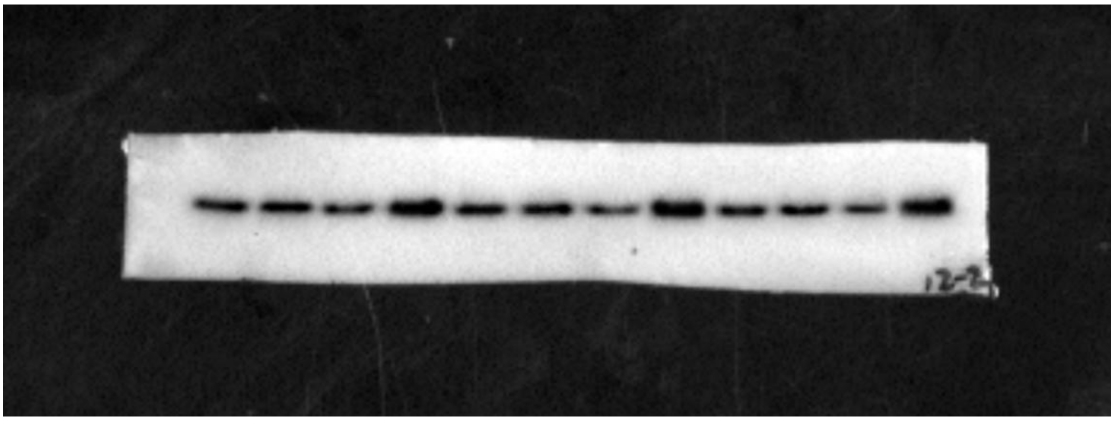

Figure 3C- pellet

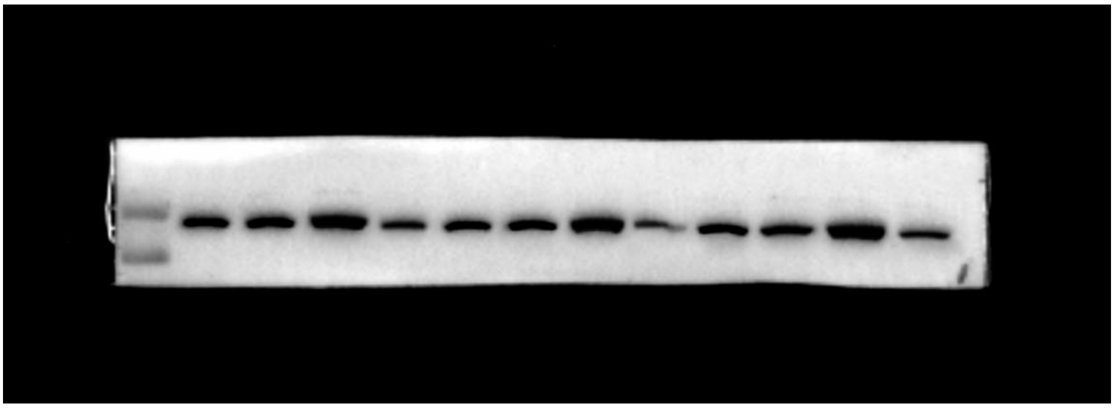

Figure 3C- soluble

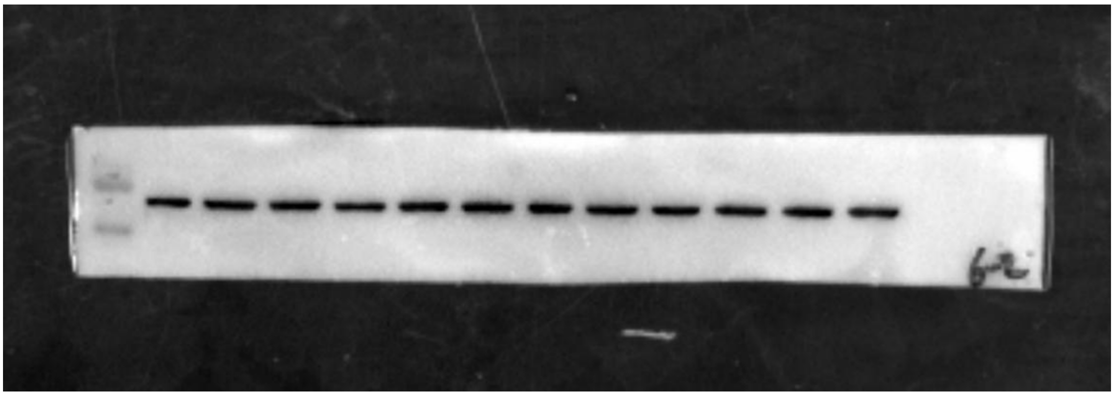

Figure 3C- whole

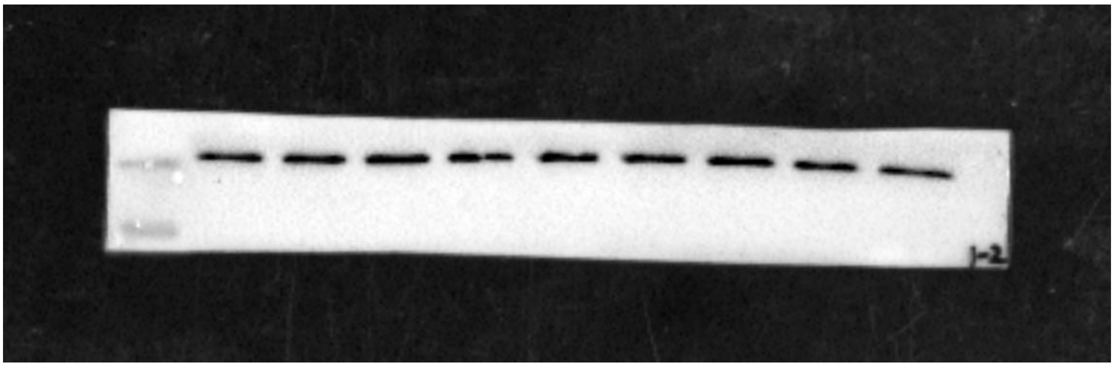

Figure 3E- input-GFP

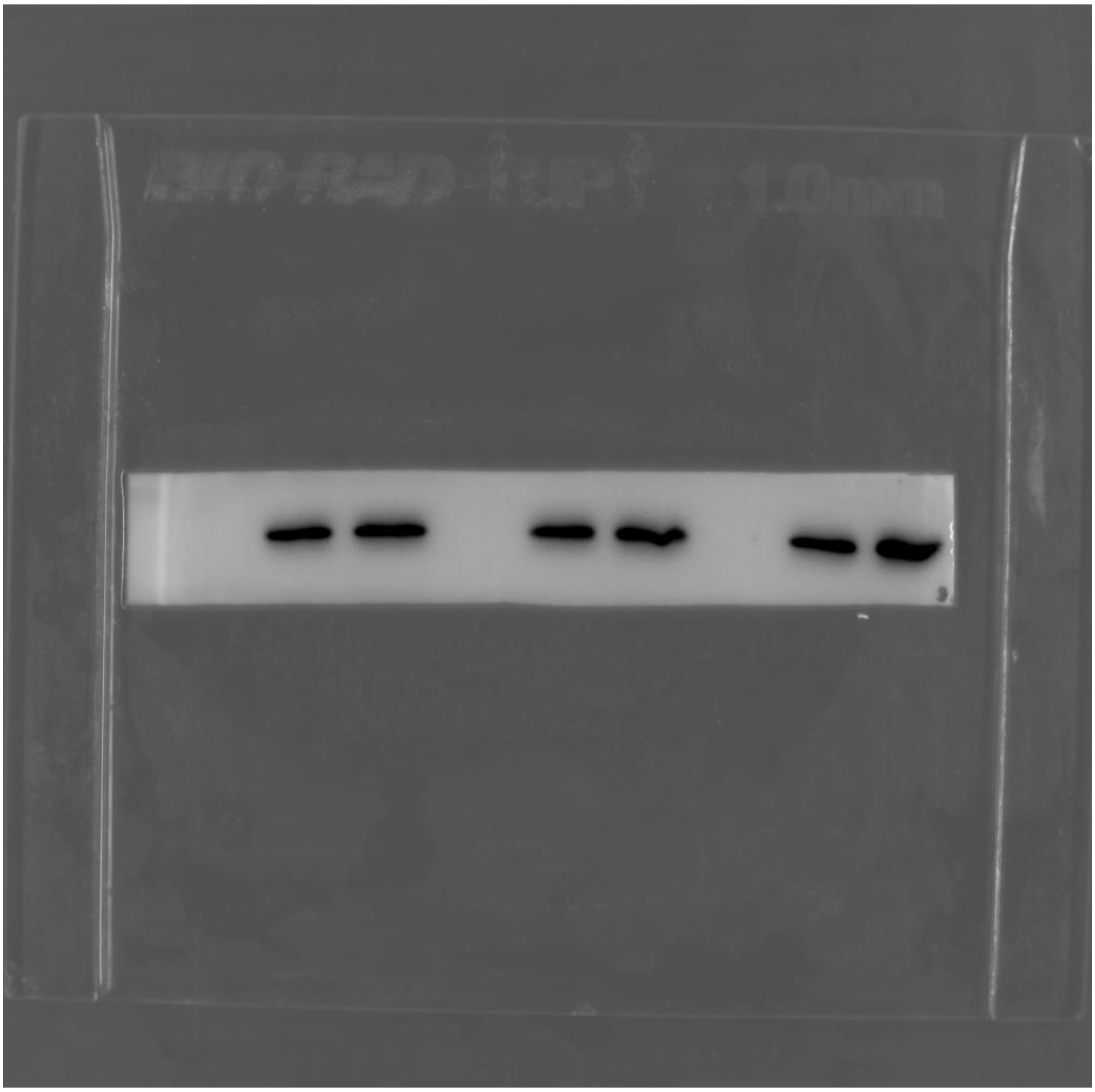

Figure 3E- input-V5

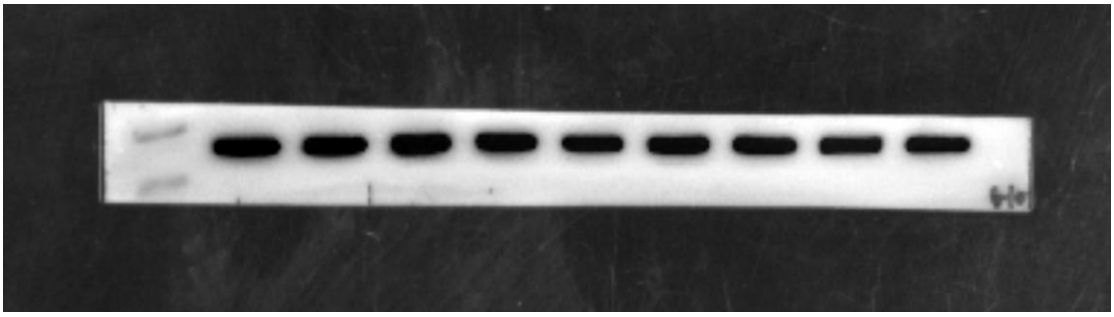

Figure 3E- input-β-actin

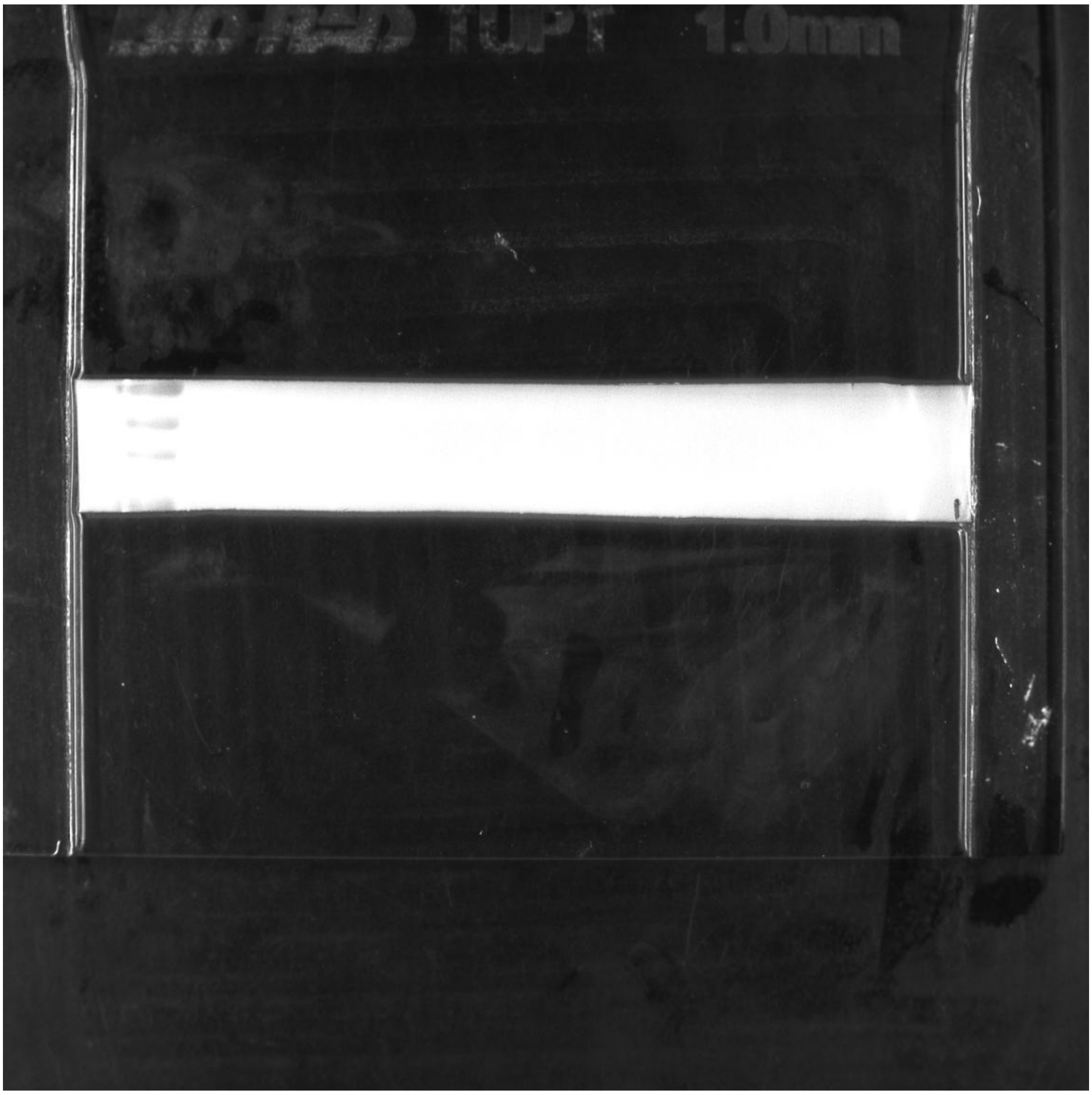

Figure 3E- IP-IgG

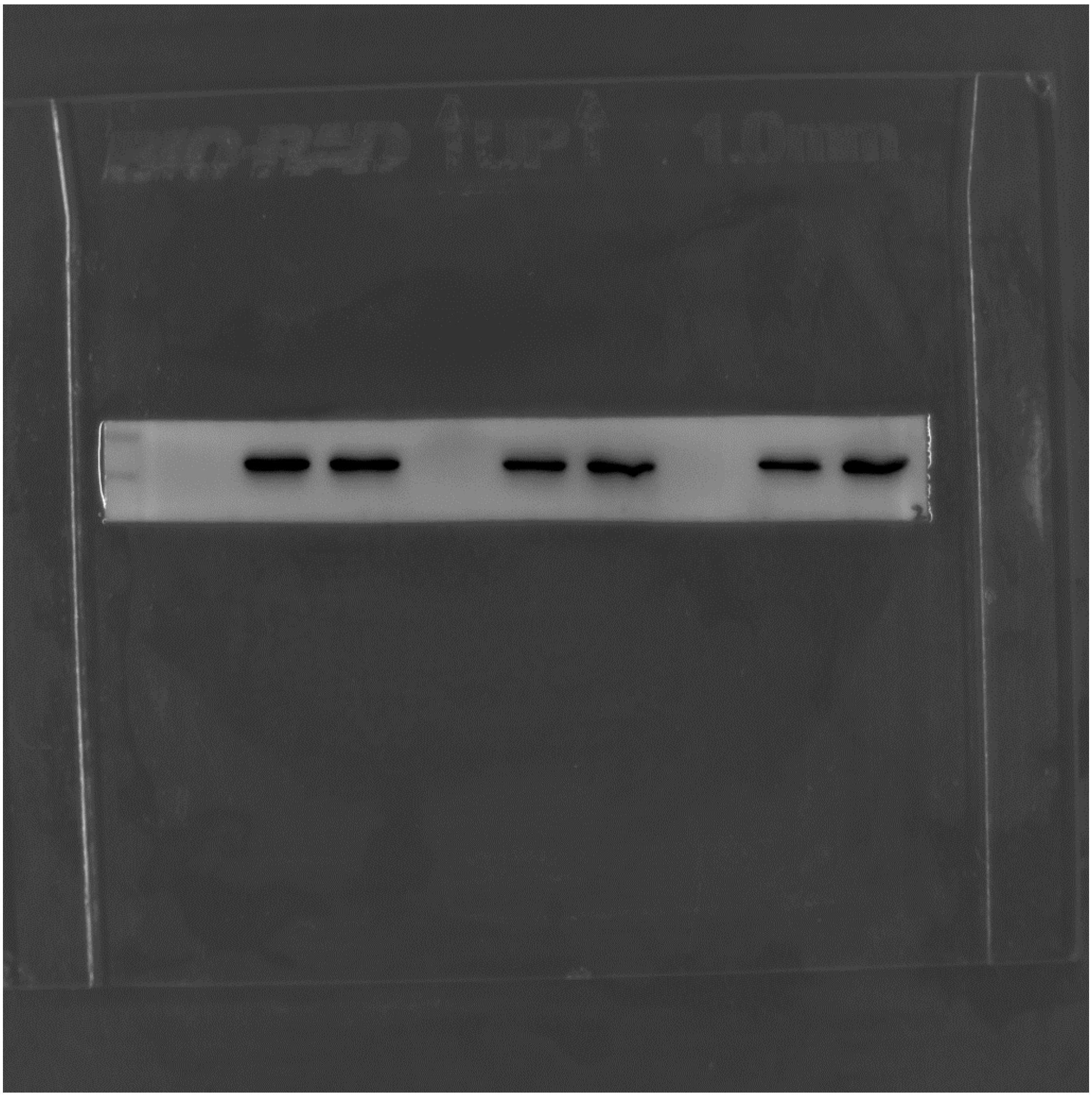

Figure 3E- IP-V5
